# Prospective associations of neighborhood healthy food access and walkability with weight status in a regional pediatric health system

**DOI:** 10.1186/s12966-023-01514-1

**Published:** 2023-09-20

**Authors:** Qianxia Jiang, Bethany Forseth, Lauren Fitzpatrick, Helena H. Laroche, Sarah Hampl, Ann M. Davis, Chelsea Steel, Jordan Carlson

**Affiliations:** 1 Center for Children’s Healthy Lifestyles & Nutrition, 610 E. 22Nd Street, Kansas City, MO 64108 USA; 2 Department of Pediatrics, Children’s Mercy Hospital, Kansas City, MO USA; 3 School of Medicine, University of Missouri Kansas City, Kansas City, MO USA; 4 University of Kansas Medical Center, Kansas City, KS USA

**Keywords:** Food access, Walkability, Childhood obesity, Built environment

## Abstract

**Background:**

Most neighborhood food and activity related environment research in children has been cross-sectional. A better understanding of prospective associations between these neighborhood environment factors and children’s weight status can provide stronger evidence for informing interventions and policy. This study examined associations of baseline and changes in neighborhood healthy food access and walkability with changes in children’s weight status over 5 years.

**Methods:**

Height, weight, and home address were obtained for 4,493 children (> 75% were Black or Latinx) from primary care visits within a large pediatric health system. Eligible participants were those who had measures collected during two time periods (2012–2014 [Time 1] and 2017–2019 [Time 2]). Data were integrated with census tract-level healthy food access and walkability data. Children who moved residences between the time periods were considered ‘movers’ (*N* = 1052; 23.4%). Mixed-effects models, accounting for nesting of children within census tracts, were conducted to model associations of baseline and changes in the neighborhood environment variables with Time 2 weight status (BMIz and overweight or obese vs. healthy weight). Models adjusted for weight status and child and neighborhood sociodemographics at baseline.

**Results:**

Children living in a neighborhood with [ample] healthy food access at Time 1 had a lower BMIz at Time 2, regardless of mover status. A decrease in healthy food access was not significantly associated with children’s weight status at Time 2. Baseline walkability and improvements in walkability were associated with a lower BMIz at Time 2, regardless of mover status.

**Conclusions:**

Findings provide evidence that residing in a neighborhood with healthy food access and walkability may support a healthy weight trajectory in children. Findings on changes in the neighborhood environment suggested that improved walkability in the neighborhood may support children’s healthy weight. The greater and more consistent findings among movers may be due to movers experiencing greater changes in neighborhood features than the changes that typically occur within a neighborhood over a short period of time. Future research is needed to investigate more robust environmental changes to neighborhoods.

## Introduction

Childhood obesity is a serious health problem in theU.S. that has substantial consequences for both the physical and mental health of children [1, 2]. The prevalence of obesity among 6-to-19 year-olds was 20.7%—22.2% in 2017–2020 [3]. There is growing attention to the potential role of neighborhood built environment characteristics in the prevention and control of childhood obesity [4, 5].

The ways communities are designed and maintained can facilitate or inhibit residents’ access to health promoting opportunities. This includes neighborhood environment characteristics like walkability and sidewalks, greenness, and access to food stores and physical activity facilities [6]. Neighborhood food environments consist of both healthy and unhealthy food retail establishments. Some researchers have observed that the availability of supermarkets or large grocery stores providing a variety of healthy foods has a preventive association with childhood obesity [7, 8]. While some studies have failed to show significant associations between neighborhood supermarket availability and children’s weight status, such associations have been more common among children from lower income families or neighborhoods [9]. Neighborhood walkability, the extent to which the neighborhood design supports walking for purposes such as transport and recreation, has been associated with greater physical activity and a healthier weight status in children [10, 11]. Studies have found age differences in the association between neighborhood walkability and physical activity, particularly that adolescents benefited more from walkable neighborhood environments than younger children [12, 13]. However, there have been limited studies spanning multiple age groups [12]. Additionally, most research has been cross-sectional. More prospective studies are needed to provide stronger evidence to inform interventions and policy [7, 8, 12].

Because the environmental changes neighborhoods experience over a relatively short time (e.g., a few or several years) are typically small, outside of studies that purposefully select areas known to be undergoing rapid and major changes (e.g., natural experiments), it is important to investigate environmental changes experienced by children who move residences. Movers can experience a range of environmental changes, including worsening, similar, and large improvements in healthy food access and walkability. A previous study observed increases in physical activity among adolescents from Army families who relocated to a station with a greater number of environmental opportunities for physical activity as opposed to fewer opportunities [14]. However, there is a lack of evidence on neighborhood environment changes and weight status in children who have moved, and studies have generally not examined both movers and non-movers.

The purpose of the current study was to examine prospective associations of baseline as well as changes in neighborhood healthy food access and walkability with children’s weight status among movers and non-movers. We hypothesized that greater access to healthy foods and greater walkability would relate to healthier trajectories in children’s weight status among both groups, with stronger associations observed among movers due to experiencing larger changes in neighborhood features.

## Methods

### Participants

The current study included 4493 children who visited a Children’s Mercy (Kansas City, MO, USA) pediatric primary care clinic during 2012–2014 (Time 1) and again during 2017–2019 (Time 2). Children were excluded from present analyses if they were not 6 to 15 years of age at Time 1, lived outside of the largest and most central 6 counties in the Kansas City metropolitan area (Cass County, MO; Clay County, MO; Jackson County, MO; Platte County, MO; Johnson County, KS; and Wyandotte County, KS), or had more than two different addresses across both time periods. See Appendix 1 for the detailed exclusion criteria. Children were grouped by their move status. ‘Movers’ had a different address during each time period, at least one visit at Time 1, and two visits at Time 2 (only the second visit was used in the analyses). ‘Non-movers’ had the same home address during both time periods. The mover and non-mover samples comprised 1052 and 3441 children across 256 and 379 census tracts across time periods, respectively. 36.9% of movers stayed in the same census tract. The average size of the included census tracts was 4.6 (SD = 13.0) square miles. The study was approved by the Children’s Mercy Institutional Review Board.

### Measures

#### Child sociodemographic characteristics and home address

Time 1 sociodemographic information (i.e., age, sex, race/ethnicity (non-Hispanic White, non-Hispanic Black, Hispanic, other race or ethnicity, unknown), and health insurance type (private, government/public, or none) came from the electronic health record (EHR). Children’s home addresses were obtained from the EHR, geocoded in ArcGIS, and aggregated at the census tract level using the Python GeoPandas library.

#### Weight status

Height and weight were measured by clinic staff and obtained from the EHR. Body mass index (BMI) percentiles and z-scores were calculated based on children’s age and sex. Overweight was classified as a BMI ≥ 85th and < 95th percentile, and obese as a BMI ≥ 95th percentile. When children had multiple visits/records within a time period, we used the first visit at Time 1 and the last visit at Time 2.

#### Neighborhood food access

Neighborhood food access was obtained from the U.S. Department of Agriculture (USDA) food access research atlas datasets that were closest in date to Time 1 (2010) and Time 2 (2019). A low food access tract represented a tract where ≥ 500 residents, or 33% of the tract population, lived > 1 mile (urban areas) or > 10 miles (rural areas) from the nearest supermarket or large grocery store [15]. The urban and rural classification was based on the United States Department of Agriculture Rural–Urban Continuum Codes shown in Online Appendix 2 [16]. Change in healthy food access between Times 1 and 2 was categorized as change from [ample] healthy food access to low healthy food access; no change; and change from low healthy food access to [ample] healthy food access. However, due to the small proportion (2%) of children changing from low healthy food access to [ample] healthy food access, this category was grouped with the no change category.

#### Neighborhood walkability index

Neighborhood walkability was obtained from the U.S. Environmental Protection Agency National Walkability Index datasets that were closest in date to Time 1 (2013) and Time 2 (2021) [17]. The national walkability index reflected physical activity-related community design features and was defined using a weighted composite of standardized values for four component variables reflecting street connectivity, employment mix (proxy for land use mix), employment and household mix (proxy for residential density), and transit access. Each iteration (i.e., 2013 and 2021) of the walkability index ranks block groups from 0 to 20 (highest walkability) based on all block groups in the U.S [17]. The composite index was investigated rather than each individual variable based on evidence suggesting an aggregation or pattern of environmental features is needed to have meaningful impacts on health [18, 19], and on prior research that observed cross-sectional associations between this index and weight or physical activity in youth and adults [20, 21]. We first investigated the four component variables separately and the associations were similar for three (i.e., street connectivity, proxy for land use mix, proxy for residential density) of the four components. Similar to previous studies in children [20, 22], we didn’t find significant associations between transit access and children’s weight status. Therefore, we used a three component walkability index in the current study. Since the present study aimed to investigate changes in walkability over time, we needed to recreate the walkability index so that the rankings considered both time points (i.e., so that an area that experienced an increase in walkability would always have a higher ranked score, and vice versa). Following the National Walkability Index procedures [17], we computed the new walkability index by rank ordering the three component variables, using the distribution of block groups within the present sample and incorporating both time points (i.e., each block group had two values, one for each time point). The block group level data were then averaged across within each census tract to match the level available for the food access data. Because the variability in walkability was similar between the present sample and the U.S. walkability index, based on the mean and standard deviation, the values can be interpreted similarly (1–5.75 Least walkable; 5.76–10.5 Below average walkable; 10.51–15.25 Above average walkable; 15.26–20 Most walkable). Change in walkability was calculated as a continuous measure by subtracting Time 1 values from Time 2 values, and further categorized as no change or decrease based on percentiles among all children (change score < 0.4 (in the bottom 25th percentile)); small increase (change score 0.4–1.8( in the 25th to 60th percentile)); and moderate increase (change score > 1.8 (in the top 15th percentile)).

#### Neighborhood sociodemographic characteristics

Time 1 census tract sociodemographic information was obtained from the 2010–2014 American Community Survey 5-year estimates and included the percentage of non-Hispanic White, non-Hispanic Black, and Hispanic Latino residents; median annual household income.

### Analysis

Descriptive statistics were computed for all study variables. Mixed-effects regression models, accounting for nesting of children within census tracts, were conducted to model the association of Time 1 values and change values for the neighborhood environment variable, concurrently in the same model, with children’s weight status at Time 2. All models adjusted for the Time 1 BMIz and child sociodemographics. A second set of models additionally adjusted for neighborhood sociodemographics. Food access and walkability were tested in separate models. BMIz was tested in linear models and overweight or obese (versus healthy weight) in logistic models. Mover status was first explored as a moderator of the aforementioned associations using multiplicative interaction. Due to consistent evidence of moderation across most models, the models presented were conducted separately among movers and non-movers (i.e., stratified analysis). Interactions between each neighborhood environment variable and sex, age, neighborhood income, and the other neighborhood environment variable (i.e., food access X walkability) were also explored, which were conducted separately within the movers and non-movers.

## Results

Sample characteristics and neighborhood sociodemographic variables at Time 1 are presented in Table 1. About half of the children were female. 34.4% of non-movers were non-Hispanic Black, whereas 54.8% of movers were non-Hispanic Black. 48.5% of non-movers were Hispanic/Latinx, while 30.4% of movers were Hispanic/Latinx.

**Table 1:** Sample characteristics at Time 1

	Non-movers (*N* = 3441)	Movers (*N* = 1052)
Child characteristics		
Age ranges (years)	6–15	6–15
Mean age (SD)	8.70 (2.29)	8.74 (2.21)
% female	48.7%	54.9%
Race/ethnicity		
% non-Hispanic White	17.1%	14.8%
% non-Hispanic Black	34.4%	54.8%
% Hispanic/Latinx	48.5%	30.4%
% Other race/ethnicity	5.8%	6.0%
Insurance type		
% with commercial health insurance	14.9%	9.5%
% with government/public health insurance	79.7%	84.5%
% with no health insurance	5.3%	5.9%
Neighborhood sociodemographic characteristics		
Population density, tract	2806.1 (1813.8)	3091.1 (1792.4)
Total area, tract	4.6 (13.4)	2.6 (6.5)
% Non-Hispanic White	61.7 (28.9)%	53.3 (29.5)%
% Non-Hispanic Black	21.3 (25.3)%	27.1 (28.6)%
% Hispanic	11.6 (13.4)%	13.7 (15.2)%
Median household income	$55,373.7 ($29,659.9)	$46,412.8 ($21,805.3)
% Rural	7.96%	5.49%

Child weight and neighborhood environment characteristics at Time 1 and Time 2 are presented in Table 2. For both groups, about 19% of children were overweight during each time period. The proportion of children with obesity was higher at Time 2 and somewhat higher among movers. For non-movers, the proportion of census tracts classified as having low healthy food access was 44.1% at Time 1, which increased slightly to 45.4% at Time 2. The average walkability index was 10.1 at Time 1 and saw a marginal increase of 1.4 point to 11.5 at Time 2. Among movers, 43.8% of census tracts were identified as having low healthy food access at Time 1, and this percentage rose to 45.3% at Time 2. The mean walkability index exhibited a similar pattern, starting at 9.6 at Time 1 and rising by 1.4 points to 11.0 at Time 2. Movers were slightly more likely to experience a decrease in healthy food access than non-movers.

**Table 2:** Child weight and neighborhood built environment characteristics at time 1 and time 2

	Time 1		Time 2	
	Non-movers (*n* = 3441)	Movers (*n* = 1052)	Non-movers (*n* = 3441)	Movers (*n* = 1052)
Child weight				
Mean (SD) BMIz	0.72 (1.11)	0.81 (1.05)	0.85 (1.14)	0.93 (1.07)
Mean (SD) BMI %ile	68.95 (28.45)	71.72 (26.44)	71.81 (28.55)	74.3 (26.51)
% overweight	18.7%	19.0%	18.8%	19.0%
% obese	22.9%	24.1%	28.4%	29.7%
Neighborhood environment characteristics				
% Low healthy food access	44.1%	43.8%	45.4%	45.3%
Walkability index [0–20]	10.1 (3.2)	9.6 (2.6)	11.5 (3.3)	11.0 (2.3)
Change in healthy food access				
% From [ample] access to low access	-	-	6.3%	11.4%
% No change	-	-	90.9%	86.7%
% From low access to [ample] access	-	-	2.7%	1.9%
Changes in walkability index, Mean (SD)	-	-	1.39 (1.61)	1.33 (2.45)
Changes in walkability index^a^				
% No change or decrease	-	-	24.9%	27.9%
% Small increase	-	-	35.5%	31.3%
% Moderate increase	-	-	39.6%	40.8%

^a^Three categories of walkability index change were based on percentiles among all children, no change: < 0.4 (25th percentile); small change: 0.4 (25th percentile) -1.8 (60th percentile); moderate change: > 1.8 (60th percentile)

Among non-movers, [ample] healthy food access at Time 1 was related to a healthier BMIz trajectory between time periods, though this association was non-significant after adjusting for neighborhood sociodemographic characteristics (Table 3). However, changes in healthy food access were not associated with children’s weight status trajectories. A higher walkability score at Time 1 was associated with a healthier BMIz trajectory and a lower odds of being overweight or obese at Time 2. An increase in walkability was associated with a healthier BMIz trajectory and lower odds of being overweight or obese at Time 2 in the full adjusted model. Compared to those who had a neighborhood with similar or poorer walkability at Time 2, those who experienced a moderate increase in walkability experienced a reduced BMIz by 0.06 at Time 2 in the fully adjusted models.

**Table 3:** Association between neighborhood food access and walkability and non-movers’ weight status at Time 2 (*N* = 3441)

	BMIz				Overweight or obese vs. healthy weight			
	Model 1^a^		Model 2^b^		Model 1^a^		Model 2^b^	
	B (95% CI)	P	B (95% CI)	P	OR (95% CI)	P	OR (95% CI)	P
Time 1 healthy food access								
Low access (ref)	-	-	-	-	-	-	-	-
Access	-0.04* (-0.08,.00)	.049	-0.04 (-0.08,0.004)	0.078	0.97 (0.789,1.20)	0.785	0.88 (0.71,1.08)	0.215
Change in healthy food access								
From [ample] access to low access (ref)	-	-	-	-	-	-	-	-
No change or from low access to [ample] access	0.004 (-0.08,0.09)	0.918	0.01 (-0.08,0.09)	0.853	1.13 (0.74,1.73)	0.567	1.12 (0.75,1.69)	0.575
Time 1 walkability								
Walkability index	-0.01* (-0.02,-.00)	0.027	-0.01* (-0.02,-0.0002)	0.046	0.95* (0.92,0.99)	0.017	0.95* (0.91,0.99)	0.012
Changes in walkability index (continuous)	-0.02* (-0.03,-0.005)	0.010	-0.02* (-0.04,-0.01)	0.007	0.95 (0.88,1.02)	0.158	0.93* (0.86,0.99)	0.047
Change in walkability index (categorical)								
No change or decrease (ref)	-	-	-	-	-	-	-	-
Small increase	-0.05 (-0.10,0.004)	0.070	-0.05 (-0.10,0.001)	0.058	1.07 (0.83,1.39)	0.589	1.03 (0.80,1.33)	0.810
Moderate increase	-0.06* (-0.11,-0.003)	0.037	-0.06 (-0.12,-0.01)	0.024	0.92 (0.69,1.19)	0.476	0.85 (0.65,1.11)	0.238

^a^Based on mixed-effects linear models adjusting for children’s race/ethnicity, age, and insurance type

^b^Based on mixed-effects linear models adjusting for children’s race/ethnicity, age, and insurance type, neighborhood median household income, and neighborhood race/ethnicity

^*^p < 0.05

Among movers, [ample] healthy food access at Time 1 was related to a healthier BMIz trajectory, though this association was non-significant after adjusting for neighborhood sociodemographic characteristics (Table 4). However, changes in healthy food access were not associated with children’s weight status trajectories. A higher walkability score at Time 1 was associated with lower odds of being overweight or obese at Time 2 in the fully adjusted models. An increase in walkability was associated with a healthier BMIz trajectory and lower odds of being overweight or obese at Time 2. Each 1 unit increase in walkability (about 1/3 of the standard deviation in walkability at Time 1) was associated with a 20% reduction in odds of being overweight or obese versus a healthy weight. Compared to those who moved to a neighborhood with similar or poorer walkability, those who experienced a small increase in walkability experienced a reduced BMIz by 0.14, and were 43% less likely to be overweight or obese at Time 2. Those who experienced a moderate increase in walkability experienced a reduced BMIz by 0.18 and were 58% less likely of being overweight or obese at Time 2 in the fully adjusted models.

**Table 4:** Association between neighborhood food access and walkability and movers’ weight status at Time 2 (*N* = 1052)

	BMIz				Overweight or obese vs. healthy weight			
	Model 1^a^		Model 2^b^		Model 1^a^		Model 2^b^	
	B (95% CI)	P	B (95% CI)	P	OR (95% CI)	P	OR (95% CI)	P
Time 1 healthy food access								
Low access (ref)	-	-	-	-	-	-	-	-
Access	-0.09* (-0.18,-0.01)	0.034	-0.08 (-0.17,0.01)	0.079	0.77 (0.54,1.10)	0.780	0.85 (0.58,1.23)	0.377
Change in healthy food access								
From [ample] access to low access (ref)	-	-	-	-	-	-	-	-
No change or from low access to [ample] access	-0.09 (-0.22,0.05)	0.192	-0.10 (-0.23,0.04)	0.164	0.75 (0.44,1.28)	0.295	0.69 (0.40,1.20)	0.192
Time 1 walkability								
Walkability index	-0.02* (-0.04, -0.01)	0.007	-0.03* (-0.04,-0.01]	0.004	0.93 (0.87,1.00)	0.054	0.91* (0.84,0.98)	0.010
Changes in walkability index (continuous)	-0.05* (-0.08,-0.02)	0.001	-0.05* (-0.08,-0.02)	0.001	0.83* (0.74,0.94)	0.004	0.80* (0.70,0.91)	0.001
Change in walkability index (categorical)								
No change or decrease (ref)	-	-	-	-	-	-	-	-
Small increase	-0.13* (-0.24,-0.03)	0.011	-0.14* (-0.24,-0.04)	0.009	0.67 (0.35,1.26)	0.212	0.57* (0.37,0.90)	0.015
Moderate increase	-0.17* (-0.28, -0.07)	0.001	-0.18* (-0.29,-0.08)	0.001	0.36* (0.18,0.74)	0.005	0.42* (0.26,0.567)	< 0.001

^a^Based on mixed-effects linear models adjusting for children’s race/ethnicity, age, and insurance type

^b^Based on mixed-effects linear models adjusting for children’s race/ethnicity, age, and insurance type, neighborhood median household income, and neighborhood race/ethnicity

^*^p < 0.05

The interaction tests revealed no significant moderation in associations between the neighborhood environment variables and weight status by sex, age, neighborhood, and income. Additionally, no interactions were detected between food access and walkability.

## Discussion

This study is among the first to explore prospective associations of changes in neighborhood food access and walkability with children’s weight status among both movers and non-movers in a large and racially and ethnically diverse sample of children. We found some evidence in support of a small association between better food access at baseline and a healthy weight trajectory over time, but no association of decreases in healthy food access over time with weight trajectories. More robust associations were observed for walkability, with greater walkability at baseline as well as improvements in walkability over time being related to healthier weight trajectories. Overall, findings suggest access to healthy foods and walkable neighborhoods are important targets for reducing population levels of overweight and obesity in children, including those living in economically disadvantaged communities.

Healthy food outlets such as grocery stores are posited to promote a healthy weight in children by providing access to affordable healthy foods and supporting healthy dietary habits [23]. Consistent with some previous studies [5, 24], we found that children living near a supermarket or large grocery store had smaller increases in BMIz over a ~ 5 yr period relative to those living further from these types of healthy food outlets. These associations were small, a difference of 0.04–0.09 BMIz (~ 1.2 – 2.7 BMI%ile) between the two groups but could be meaningful at the population level given almost half of the sample had low healthy food access. In the current study, children who experienced a decrease or no/little change in healthy food access experienced similar changes in weight status over time. One possible explanation for the association of baseline healthy food access but not change in healthy food access with weight status is that food purchasing may be influenced by prior habits, as well as by the dynamics of other neighborhood food outlets [5]. For example, those used to purchasing healthy foods from a grocery store may be more willing to travel further to continue these habits, when possible. Another potential explanation for the lack of association for change in healthy food access is that very little change was observed, which made it necessary to group children who experienced a change from low healthy food access to [ample] access with those who did not experience changes in healthy food access.

Living in a more walkable environment has been shown to promote physical activity and discourage a sedentary lifestyle, ultimately reducing risk of being overweight or obese [10, 25, 26]. The present study extends previous research on walkability and childhood obesity, most of which has been cross-sectional [26], by presenting prospective associations. The findings suggest improved walkability in a neighborhood may provide some protection against the increases in BMIz and obesity risk children typically experience as they become older and transition to adolescence. The magnitudes of association appear clinically meaningful. Each increase in walkability by 1 standard deviation (3 units on the 1–20 walkability scale) related to ~ 21% reduction in the odds of being overweight or obese among non-movers, and ~ 60% among movers. The magnitude of association for BMIz as a continuous dependent variable, which was significant, indicated each increase in walkability by 1 standard deviation related to lower BMIz by 0.06 (2 BMI %ile) among non-movers, and 0.15 (5.1 BMI %ile) among movers. The greater and more consistent findings among movers may have been due to movers experiencing larger changes or improvements in walkability (i.e., greater variability), given that changes occurring within a neighborhood over a short period of time are typically small. This is in line with previous research that shows an accumulation of features has the greatest potential to support physical activity and health [18, 27–29]. In accordance with prior research, street connectivity and residential density may be the most important macro-level walkability features for children’s physical activity and weight status, whereas walkability features like mixed land use and transit access may be more important for adults than children [12, 13, 20]. Research using natural experiment designs to focus on neighborhoods experiencing large and robust improvements in walkability are important for providing more insight into these findings.

### Limitations and future directions

One limitation is that the use of census tract level measures does not accurately reflect the environments experienced by all residents across the tract, and these boundaries may not match individuals’ perceptions of their neighborhood. Additionally, children’s weight status can be influenced by various factors beyond the scope of the current study (e.g., home environment, school environment, other aspects of the neighborhood environment). The food access and walkability measures were limited to broad macro-scale community design features and did not include micro-scale features such as the availability, quality, and cost of healthy foods within grocery stores or the presence of pedestrian safety and other streetscape features [30, 31]. The presence of unhealthy food options was also not assessed, which could be accomplished in future research by integrating detailed food retail establishment data to more precisely measure the availability and locations of various food outlets in a given area (e.g., with walkable or drivable distance among each resident). We were unable to determine how long the movers lived at their new address, and although we excluded those with only one well child visit while at their new address, the potential duration of exposure at the new address was brief, likely resulting in 1.5 to 3 years on average. Because of the constraints of utilizing medical records, the lack of measures related to children’s diet and physical activity, and the locations in which these things occur, hindered our ability to investigate the behavioral mechanisms that are posited to link these neighborhood environment characteristics with childhood obesity. Since the reason for moving was now known, selection bias could have driven the observed associations. More research on whether and how healthy food access and walkability impact neighborhood selection is needed to inform the generalizability of observational findings. The current study was conducted in a specific geographic area and within a single, albeit large, health system. Thus, the findings may not generalize to different regions or populations. The exclusion of children who moved outside of the Kansas City region may also impact the study’s generalizability.

## Conclusions

Overall, some support was provided for the hypothesis that access to healthy foods is associated with smaller (i.e., healthier) increases in weight status as children become older, and greater support was provided for the hypothesis related to walkability, neighborhood with improved walkability may be associated with smaller increases in weight status as children become older. Future research is needed to evaluate whether children can experience bigger benefits when robust improvements are made to a neighborhood’s walkability. Urban planner or developers should try to consider the potential role of the neighborhood environment in supporting children’s healthy eating, and active living, and health.

## Figures and Tables

**Fig. 1:**
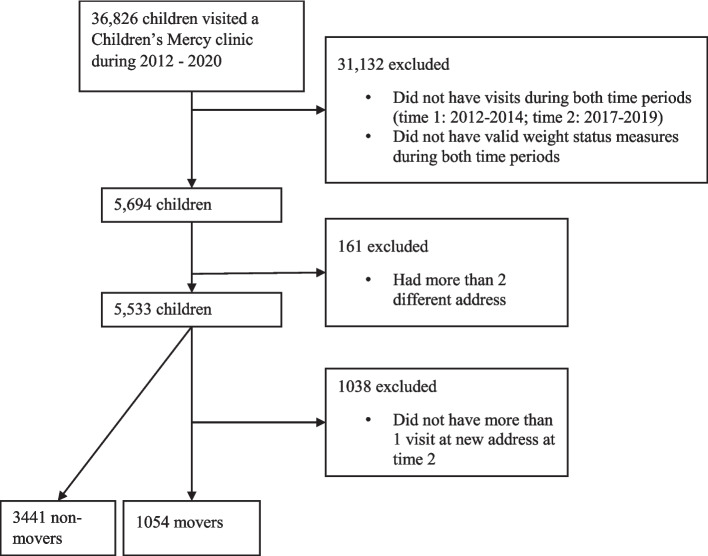
Sample selection diagram

## Data Availability

The data that support the findings of this study are available from the author Carlson on reasonable request.

## Appendix 2

**Table 5:** Categories for metro and non-metro counties

Code	Description
Metro counties	
1	Counties in metro areas of 1 million population or more
2	Counties in metro areas of 250,000 to 1 million population
3	Counties in metro areas of fewer than 25,000 population
Non-metro counties	
4	Urban population of 20,000 or more, adjacent to a metro area
5	Urban population of 20,000 or more, not adjacent to a metro area
6	Urban population of 2,500 to 19,999, adjacent to a metro area
7	Urban population of 2,500 to 19,999, adjacent to a metro area
8	Completely rural or less than 2,500 urban population, adjacent to a metro area
9	Completely rural or less than 2,500 urban population, not adjacent to a metro area

## Abbreviations

HER: Electronic Health Record
BMI: Body mass index
USDA: The U.S. Department of Agriculture
